# Telomerase Inhibitor Imetelstat (GRN163L) Limits the Lifespan of Human Pancreatic Cancer Cells

**DOI:** 10.1371/journal.pone.0085155

**Published:** 2014-01-07

**Authors:** Katrina M. Burchett, Ying Yan, Michel M. Ouellette

**Affiliations:** 1 Eppley Institute for Research in Cancer, University of Nebraska Medical Center, Omaha, Nebraska, United States of America; 2 Department of Biochemistry and Molecular Biology, University of Nebraska Medical Center, Omaha, Nebraska, United States of America; Tulane University Health Sciences Center, United States of America

## Abstract

Telomerase is required for the unlimited lifespan of cancer cells. The vast majority of pancreatic adenocarcinomas overexpress telomerase activity and blocking telomerase could limit their lifespan. GRN163L (Imetelstat) is a lipid-conjugated N3′→P5′ thio-phosphoramidate oligonucleotide that blocks the template region of telomerase. The aim of this study was to define the effects of long-term GRN163L exposure on the maintenance of telomeres and lifespan of pancreatic cancer cells. Telomere size, telomerase activity, and telomerase inhibition response to GRN163L were measured in a panel of 10 pancreatic cancer cell lines. The cell lines exhibited large differences in levels of telomerase activity (46-fold variation), but most lines had very short telomeres (2–3 kb in size). GRN163L inhibited telomerase in all 10 pancreatic cancer cell lines, with IC_50_ ranging from 50 nM to 200 nM. Continuous GRN163L exposure of CAPAN1 (IC_50_ = 75 nM) and CD18 cells (IC_50_ = 204 nM) resulted in an initial rapid shortening of the telomeres followed by the maintenance of extremely short but stable telomeres. Continuous exposure to the drug eventually led to crisis and to a complete loss of viability after 47 (CAPAN1) and 69 (CD18) doublings. Crisis In these cells was accompanied by activation of a DNA damage response (γ-H2AX) and evidence of both senescence (SA-β-galactosidase activity) and apoptosis (sub-G1 DNA content, PARP cleavage). Removal of the drug after long-term GRN163L exposure led to a reactivation of telomerase and re-elongation of telomeres in the third week of cultivation without GRN163L. These findings show that the lifespan of pancreatic cancer cells can be limited by continuous telomerase inhibition. These results should facilitate the design of future clinical trials of GRN163L in patients with pancreatic cancer.

## Introduction

Pancreatic cancer is the fourth leading cause of cancer death in the Western world. Pancreatic cancer is a disease of insidious progression and high lethality, with a 5-year survival rate of just 6%. In the United States alone, an estimated 43,920 patients are expected to be diagnosed with the disease in 2012, and 37,390 patients are expected to die from it [1]. The vast majority of these cases are pancreatic ductal adenocarcinomas, which develop in the ducts of the pancreas. These highly invasive tumors consist of an abundant desmoplastic stroma, in which are embedded malignant cancer cells expressing markers of pancreatic ductal cells [2], [3]. For patients with pancreatic ductal adenocarcinoma, the only curative option is surgery [3]. The standard procedure is a pancreaticoduodenectomy (or Whipple procedure), a surgical operation that removes the head of the pancreas but spares the remaining tissue. Unfortunately, most pancreatic cancer patients present with unresectable metastatic or locally advanced disease. In fact, only 20% of patients have resectable tumors at the time of diagnosis [3]. But even for those patients who undergo surgery, the overall 5-year survival rate is of just 20%, as most of these patients will relapse within a year of their surgery [3]. Hence, there is a critical need for novel drugs that can more efficaciously target these tumor cells and/or reduce the incidence of recurrence. Telomerase inhibitors have been proposed to be especially well-suited to block the regrowth of residual cancer cells after conventional cancer therapy [4], [5]. Not only do they selectively target the telomerase-positive cancer cells, but their growth inhibitory effects increase as the targeted cells perform an increasing number of cell divisions. In the present study, we have characterized the effects of a telomerase inhibitor, GRN163L, on the cellular lifespan and survival of a panel of pancreatic cancer cell lines.

Telomerase is the enzyme responsible for the maintenance of telomeres, essential structures that cap and protect the ends of linear chromosomes. Human telomeres are made of tandem copies of (TTAGGG)_n_ DNA repeats and of associated proteins, which together form a protective capping complex [6], [7]. This cap protects chromosomal ends from degradation, interchromosomal fusions and from being recognized as double-stranded (ds) DNA breaks, a form of DNA damage [7], [8]. Because of problems associated with the replication of the ends of linear DNA molecules, the so-called end-replication problems, telomeres shorten each time human somatic cells divide and this attrition limits their lifespan [9]. Once the shortest telomere become uncapped, a DNA damage response is induced that mobilizes the p53 and p16/pRB pathways, which then act together to induce senescence, a viable state of irreversible quiescence [10], [11]. If the p53 and p16/pRB pathways are disabled, the cells will ignore these growth inhibitory signals and will continue to divide and shorten their telomeres. Eventually, terminal telomere shortening lead to crisis, a non-viable state associated with programmed cell death [10], [11]. Crisis is triggered by recurrent cycles of telomere-telomere fusions, anaphase bridges and chromosome breakage [12]. When present, telomerase can prevent the induction of senescence and crisis and extend cellular lifespan by the synthesis and addition of new telomeric repeats to the telomeres. Telomerase is ubiquitously present in the early stages of human development. But by the time of birth, expression of the enzyme is repressed and telomerase becomes absent from most somatic tissues [13], [14], including the pancreas [15], [16], [17]. Cancer specimens, in stark contrast to normal tissues, are almost always positive for telomerase activity [18], including pancreatic ductal adenocarcinomas [15], [16], [17]. Detected in more than 85% of cancers, irrespective of the tumor type, telomerase is one of the best known markers of cancer cells [18]. Moreover, this expression of telomerase in cancer cells is required for their unlimited proliferation or immortality, a hallmark of cancer. Accordingly, the inhibition of telomerase in cancer cells leads to telomere attrition and limits the lifespan of these cells [19], [20], [21], [22]. After sufficient telomere attrition has taken place, telomerase-inhibited cancer cells will succumb to either senescence or apoptosis, depending on the cellular system. This reliance on telomerase from their unlimited growth and the almost universal expression of telomerase in cancer cells make telomerase an attractive target for cancer therapy. A potential drawback, however, are the delays needed before the targeted cancer cells have lost sufficient telomeres for senescence or crisis to be induced. This delayed action might preclude their use as a first line of treatment for cancer, but to block the regrowth of residual disease after conventional therapy, telomerase inhibitors have been expected to have good therapeutic potential [4], [5].

Human telomerase contains two essential subunits: the protein hTERT (human Telomerase Reverse Transcriptase) and the small nuclear RNA hTR (human Telomerase RNA). The first provides catalytic activity and the second contains a short sequence (5′-CUAACCCUAA-3′) that serves as a template for the synthesis of telomeric repeats [23]. The substrate of telomerase is the single-stranded 3′-telomeric overhang that caps the very end of all telomeres. The enzyme functions as a reverse transcriptase and uses the RNA hTR as a template for the synthesis and addition of telomeric DNA repeats to the 3′-telomeric overhangs. For telomerase inhibition, the template region of the human telomerase RNA (hTR) presents an accessible target for oligonucleotide-based inhibitors [4], [24]. Oligonucleotides designed to hybridize to the template region can be used to inhibit the activity of telomerase [25], [26]. Oligonucleotides with various chemistries have been tested and N3′-P5′ thio-phosphoramidate oligonucleotides have yielded some of the most potent and selective inhibitors of telomerase [25], [27], [28]. These compounds are non-toxic, water soluble, nuclease resistant and display high thermal stability of duplexes formed with their complementary RNA strands. GRN163L is a second generation N3′-P5′ thio-phosphoramidate telomerase inhibitor designed by Geron Corp. (Menlo Park, CA) [20]. This inhibitor, also known as Imetelstat, carries a 5′-terminal palmitoyl moiety conjugated to the N3′>P5′ thio-phosphoramidate backbone (5′-palmitate-TAGGGTTAGACAA-NH_2_-3′). GRN163L is lipid soluble and does not require transfection for cellular uptake. At nanomolar concentrations, GRN163L inhibits telomerase in a large spectrum of cancer cell lines [20]. In follow-up studies, long term GRN163L exposure could limit the lifespan of cultivated cancer cells derived from glioblastoma [29], multiple myeloma [30] and Barrett’s esophageal adenocarcinoma [31] as well as breast [32], [33], lung [34] and liver [35] cancers. In mouse models, the inhibitor could inhibit the growth of xenografts produced in mice by the implantation of these human cancer cells [29], [30], [31], [33], [34], [35]. GRN163L is currently in clinical trials in patients with multiple myeloma (ClinicalTrials.gov Identifier: NCT01242930), essential thrombocythemia or polycythemia vera (NCT01243073), and primary or secondary myelofibrosis (NCT01731951).

The effects of telomerase inhibition, let alone GRN163L, have never been examined in pancreatic cancer, one of the deadliest and most frequently recurring malignancies. In this report, we have tested the effects of GRN163L on a panel of 10 pancreatic cancer cell lines. With IC_50_ in the nanomolar range, GRN163L efficaciously inhibited telomerase in all 10 cell lines. Continuous GRN163L exposure of CAPAN1 and CD18 cells resulted in an initial rapid shortening of the telomeres followed by the maintenance of extremely short but stable telomeres. Continuous exposure to the drug eventually led to crisis and to a complete loss of viability after 47 and 69 doublings, respectively. These results show that continuous exposure to GRN163L can reverse the immortal phenotype of pancreatic cancer cells. These findings should facilitate the design of future clinical trials of GRN163L in patients with pancreatic cancer.

## Materials and Methods

### Materials

Fetal bovine serum (FBS) was from HyClone (Logan, UT, USA). T4 polynucleotide kinase, Proteinase K, Gentamycin, Penicillin/Streptomycin, Dulbecco’s modified Eagle’s medium (DMEM), RPMI-1640 medium, Iscove’s modified Dulbecco’s medium and McCoy’s 5A medium were purchased from Invitrogen Corp. (Carlsbad, CA, USA). Restriction enzymes AluI, MspI, HaeIII, HinfI and RsaI were from New England BioLabs (Ipswich, MA, USA) whereas CfoI was obtained from Promega Corporation (Madison, WI, USA). The mammalian protease Inhibitor cocktail was from Sigma-Aldrich (Saint-Louis, MO, USA). The palmitoyl-conjugated N3′>P5′ thio-phosphoramidate GRN163L oligo (5′-palmitate-TAGGGTTAGACAA-NH_2_-3′) and mismatched oligo (5′-palmitate-TAGGTGTAAGCAA-NH_2_-3′; mismatches underlined) were provided by Geron Corporation (Menlo Park, CA, USA). The N3′>P5′ thio-phosphoramidate oligos were dissolved in phosphate-buffered saline (PBS) to make 1 mM stocks, which were kept frozen at −80°C. All other chemicals were from Fisher Scientific (Pittsburgh, PA, USA).

### Cell Lines

Cell lines used were previously described by others [36], [37], [38], [39], [40], [41]. Cells were maintained at 37°C in a humidified atmosphere containing 5% CO_2_. All cell lines were cultivated in medium supplemented with 10% FBS and either 50 µg/ml gentamycin or 100 U/ml penicillin/streptomycin. Media used were as follows: DMEM for most cell lines (VA13, HeLa, HPAF, CD18, Hs766T, CAPAN1, MiaPaCa2, Panc1 and L3.6pl cells); RPMI-1640 medium for AsPC1 cells; Iscove’s modified Dulbecco’s medium for CFPAC1; and McCoy’s 5A medium for CAPAN2 cells.

### Telomere Size Analysis

Total genomic DNA was isolated from frozen pellets of cells. Briefly, cells were resuspended in 2 volumes of a buffer containing 100 mM NaCl, 10 mM Tris-HCl (pH 8.0) and 25 mM EDTA (pH 8.0), after which 0.4 mg/ml Proteinase K was added. Sodium dodecyl sulfate (SDS) was then added to a final concentration of 0.5% (w/v) and the samples were rotated overnight at 50°C. The next day, samples were extracted twice with Tris buffer-saturated phenol and then once with water-saturated CHCl_3_. Next, samples were dialyzed overnight at 4°C against two changes of TE buffer (1 mM EDTA and 10 mM Tris-HCl, pH 8.0). Genomic DNA samples were stored at 4°C.

Genomic DNA (5–10 µg) was digested at 37°C for 2–4 hours in 100 µl of React 1 buffer (10 mM MgCl_2_ and 50 mM Tris-HCl, pH 8.0) with a cocktail of restriction enzymes that fail to cut telomeric DNA: AluI, CfoI, HaeIII, HinfI, MspI, and RsaI (16 units each). Digested samples were loaded on a 25 cm long 0.7% agarose gel in 0.5× TBE buffer (Tris-EDTA-Borate), along with a end-labeled [^32^P]-molecular weight DNA marker. Electrophoresis was done overnight at 50 Volts, after which the gel was transferred to a 3 M paper and vacuum-dried at 60°C for an hour. The gel was soaked in 1.5 M NaCl, 0.5 M NaOH for 15 minutes, during which the 3 M paper was peeled off. The gel was neutralized for 2×15 minutes in 1.5 M NaCl, 0.5 M Tris-HCl, pH 7.4 and then transferred to 6× SSC (750 mM NaCl, 75 mM sodium citrate, pH 7.0). Pre-hybridization was performed for 2 hours at 30°C in a buffer containing 5× SSC, 5× Denhardt’s solution, 1 mM EDTA, 0.1% SDS (w/v) and 10 mM LiCl. The hybridization was performed in the same buffer at 37°C overnight, using 0.5–1.0×10^6^ cpm/ml of an end-labeled [^32^P]-(TTAGGG)_4_ probe. The gel was pre-washed in 5× SSC (twice for 10 minutes each at 37°C) and then in 0.1× SSC containing 0.1% SDS (thrice for 10 minutes each at room temperature). Gel was blotted dry, covered with Saran Wrap and exposed to a phosphoImager cassette.

Median telomere sizes were estimated from the densitometric scanning of each lane. Signal intensity was first plotted as a function of the migration distance. Radiolabeled molecular weight markers were used to produce a calibration curve with which to recalculate size for each distance migrated. Telomere signal intensity was then replotted as a function of estimated size, rather than distance (e.g. Figure S4). Median was estimated as the size that corresponded to half the cumulative sum of all signal intensities.

### Determination of Relative Telomerase Activity

Telomerase activity was measured using a nonradioactive modification of the TRAP assay (Telomeric Repeat Amplification Protocol), according to Herbert *et al*. [42]. The assay was performed using the TRAPeze telomerase detection kit (cat # S7700; Millipore, Billerica, MA), replacing the [^32^P]-labeled TS oligo with a Cy5-conjugated TS oligo (5′-Cy5-AATCCGTCGAGCAGAGTT-3′; Integrated DNA Technologies, Coralville, IA). The assay was performed according to the manufacturer’s instruction, except that: 1) the loading dye did not contain xylene cyanol; 2) PCR was performed for 33 cycles; 3) gel was scanned directly without drying. Half reactions were separated on a 10% polyacrylamide/0.5× TBE gel for 165 minutes at 200 volts, after which scanning of the gel was performed using a Molecular Dynamics Typhoon Imager System (Molecular Dynamics). The assay incorporates an internal standard (ITAS) with which to normalize the signals for differences in PCR efficiency. With the ImageQuant program (Molecular Dynamics), telomerase activity was calculated from the ratio of the intensity of the telomeric products over that of the ITAS. To compare levels of telomerase activity between cell lines, serial dilutions (50, 100, 200, 500 cells/assay) of each line were assayed in parallel for telomerase activity (telomerase products/ITAS ratio). Plotting telomerase activity as a function of cell counts, the data from each line was fitted by least squares regression to a linear curve. The slope of each curve and 95% confidence intervals could then be used to compare relative levels of telomerase activity.

### Determination of IC_50_ for GRN163L

IC_50_ determinations were performed in 96-well plates. Cells were seeded at 5×10^4^/well, allowed to attached, and then exposed in triplicates to a variable dose of GRN163L (0, 50, 100, 200, 500, 1000, 2000, 4000 nM) or mismatched oligo (0, 4000 nM). Twenty-four hours later, cells were washed three-times with 100 µl of ice-cold phosphate-buffered saline (PBS) and then lysed in 50 µl of 1×CHAPS buffer (TRAPeze kit). Lysis was done by rocking on ice for 30 minutes, after which the 96-well plate was frozen. The day of the assay, the plate was thawed and samples were diluted 1∶20 onto a new plate using ice-cold 1×CHAPS buffer (final concentrations = 50 cells/µl). Two microliters of each diluted sample (100 cells) were then assayed as described above using the non-radioactive TRAP assay. Telomerase activity (Telomerase products/ITAS ratio) was calculated for each data point, was then expressed as a percent of the average value of the untreated samples (n = 3), and was then reported on scatter plot as a function of the log[GRN163L]. With SigmaPlot version 11.0, data points were subsequently fitted by non-linear regression to a 2-parametter sigmoid curve (Percent telomerase = D+(100–D)/(1+10^((log[GRN163L]-log[IC50])*1.59)^), where D is the minimal residual activity), from which we estimated the value and 95% confidence interval of the log[IC_50_].

### SA-β-galactosidase Activity

Cells were plated at a density of 10^4^ cells/well in a 24-well plate. The next day, cells were fixed and subjected to histochemical staining for human senescence-associated β-galactosidase activity. Fixation and staining was done as described previously [43]. Without a phase contrast filter, both the positively (blue) and negatively (unstained) stained cells were counted under the microscope. Combining data from ten separate fields, the percent of blue cells was tabulated from a total of at least 200 counted cells.

### Growth Curves

Cells were seeded at a density of 3×10^5^ cells per 150 mm dish. Drugs (PBS vehicle, 1 µM GRN163L, 1 µM mismatched oligo) were added as soon as the cells became attached (after 4–8 hours). Cells were cultivated for 7 days, during which period fresh drugs were added every 2–3 days. After a week of growth, cells were trypsinized and counted, and the number of population doublings done by each sample was re-calculated. Cells were then plated at the same original density for another week of growth, whereas remaining cells were set aside for either DNA isolation or for making frozen stocks. Cells were maintained in culture until the complete loss of the GRN163L-treated cultures.

### Western Blot Analysis

Adherent cells were washed twice with ice-cold phosphate-buffered saline (PBS), harvested by scraping in a lysis buffer containing 50 mM Tris-HCl (ph 7.5), 1 mM EDTA, 1 mM EGTA, 1% Triton X-100, 1% mammalian protease Inhibitor cocktail, and each of the following kinase/phosphatase inhibitors: 1 mM Na_3_VO_4_, 50 mM NaF, 5 mM sodium pyrophosphate, 10 mM sodium 2-glycerophosphate, and 1 µM microcystin. Floating cells were collected by centrifugation, after which cells were lysed in the same lysis buffer. Combined floating and adherent cells from the same dish were flash frozen and stored at −80°C. Proteins were quantified using the Bradford method (Bio-Rad). Samples (80–100 µg/well) were separated on 4–20% gradient SDS-PAGE gels (Bio-Rad) and transferred to nitrocellulose membranes (Bio-Rad). Blocking steps and incubations with the antibodies were done in TBS-T (50 mM Tris-HCl, 150 mM NaCl, 0.05% Tween-20, pH 7.6) containing either 5% fat-free dry milk or 5% bovine serum albumin (when using phospho-specific primary antibodies). Washes were done using TBS-T. Signals were detected using the SuperSignal West Pico kit (Thermo Scientifics). Antibodies used included against H2AX (rabbit antiserum, cat # 07-627) and γH2AX (phospho-Ser139, mouse monoclonal, clone JBW301) were from Upstate. Anti-PARP1 antibody (mouse monoclonal, clone C-2-10) was from Calbiochem/EMD Biosciences whereas the anti-actin antibody (goat polyclonal, sc-1616) was from Santa Cruz Biotechnology. Secondary antibodies used were horseradish peroxidase-conjugated antibodies against mouse, rabbit or goat IgG (Jackson ImmunoResearch).

### Flow Cytometric Analysis

Adherent cells were collected by trypsinization followed by centrifugation. Floating cells were collected by centrifugation. Combined adherent and floating cells were resuspended in PBS, fixed by the addition of ethanol to 80%, and then stored at −20°C. Twenty thousand fixed cells were stained with Propidium Iodide and analyzed for DNA content following the manufacturer’s instructions using a FACS Calibur instrument (Beckon Dickinson, Mansfield, MA, USA).

### Immunofluorescence Analysis of Telomeres

Cells grown on cover slips were washed in PBS and fixed in methanol:acetone [1∶1] at −10°C for 15 minutes. After a wash in PBS, samples were blocked at RT for 30 minutes in PBS containing 10% horse serum. After a wash in PBS, samples were incubated at 4°C overnight with the primary antibody in PBS containing 2% horse serum. After three 5 minutes washes in PBS, samples were incubated at RT for 1 hour with the secondary antibody in PBS containing 2% horse serum. After three 10 minutes washes in PBS, samples were mounted in Vectashield hard containing DAPI. Images were visualized on a Zeiss 510 Meta Confocal Laser Scanning Microscope. Primary antibodies used were a rabbit anti-TRF2 (H-300, Santa Cruz) and mouse anti-γ-H2AX antibodies (clone JBW301, Upstate). Secondary antibodies were a FITC-conjugated donkey anti-rabbit antibody and a Cy5-conjugated donkey anti-mouse antibody, both from Jackson ImmunoResearch.

### Detection of ALT by Quantitative PCR

Detection of telomeric C-rich circles indicative of ALT was performed as described by the Reddel group [44]. In triplicates, 20 µl reactions were contained 32 ng of genomic DNA, 0.2 mg/ml bovine serum albumin, 0.1% Tween-20, 4 mM dithiothreitol (DTT), 7.5 Units of Φ29 DNA polymerase (New England Biolabs, Ipswich, MA), 1× Φ29 DNA polymerase buffer and 1 mM each dATP, dCTP, dGTP and dTTP. Polymerase reactions were incubated at 30°C for 8 hours, followed by 65°C for 20 minutes. Reactions were dot blotted onto a nitrocellulose membrane (Bio-Rad). After UV-crosslinking, the membrane was hybridized overnight to a [^32^P]-labeled (TAACCC)_4_ probe. Hybridization and washes were performed as described in the Telomere size analysis section (see above).

## Results

### Telomere Length and Telomerase Activity Vary Among Pancreatic Cancer Cell Lines

**Figure 1:**
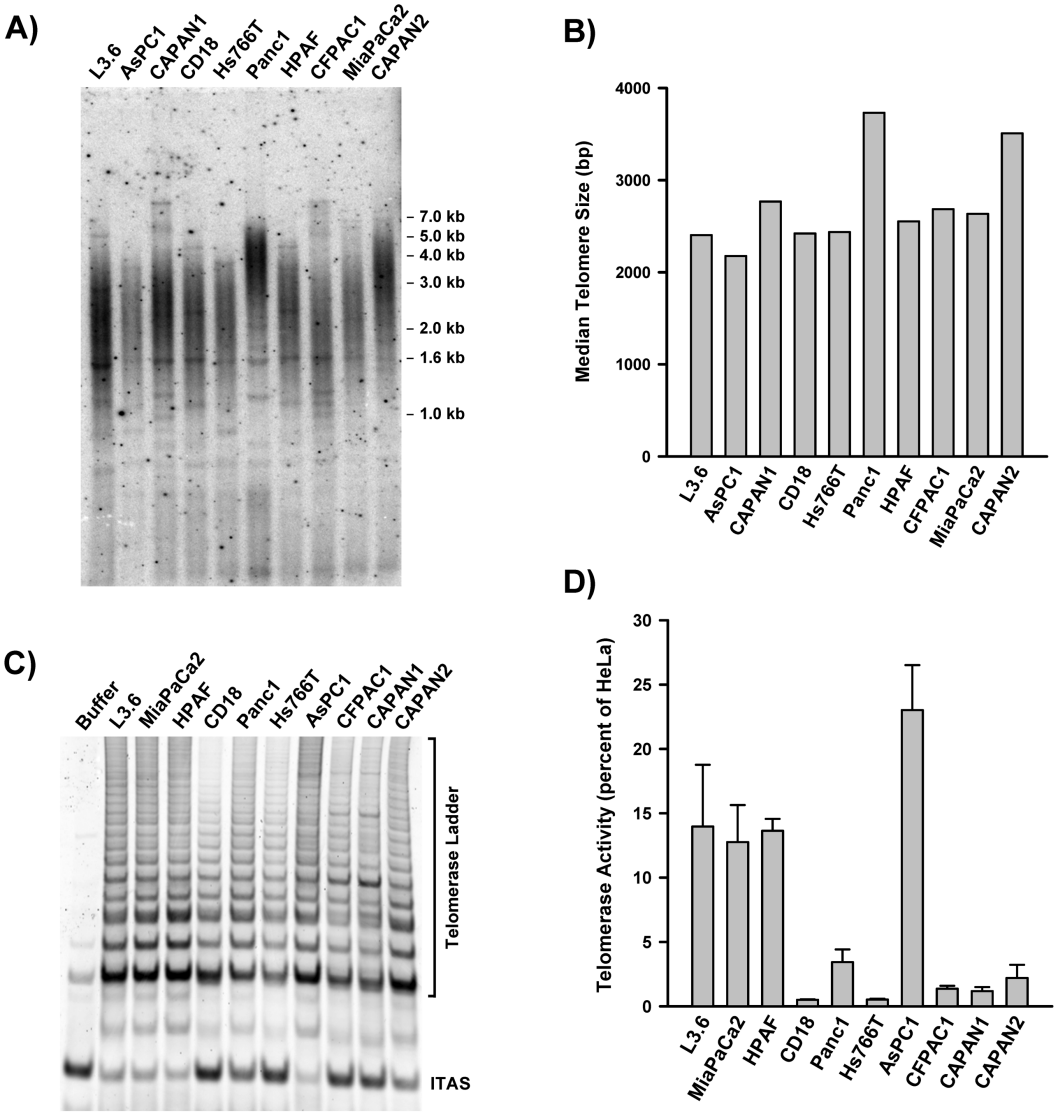
Telomere size and telomerase activity among pancreatic cancer cell lines. A) Telomere size measurements. Genomic DNA was digested with restriction enzymes, resolved by electrophoresis in agarose gels and detected by in situ hybridization to [^32^P]-(CCCTAA)_4_. B) Quantification of telomere size. Gel in A was scanned and the intensity of each lane was plotted as a function of telomere size, as described in the Material and Methods section. The median telomere size was estimated as the size corresponding to half the cumulative sum of the intensities. C) Detection of telomerase activity by the TRAP assay. Cell extracts containing 300 cells each were assayed using a Cy5-labeled TS oligo substrate. After PCR with the same oligo and a telomeric comeback primer, products were resolved by electrophoresis and detected with a Typhon PhosphoImager. Buffer was lysis buffer only. ITAS, Internal Telomerase Assay Standard. D) Quantification of relative telomerase activity. Relative telomerase activity was calculated as the ratio of the intensity of the telomerase ladder over the intensity of the ITAS. Each measurement is the Mean ± S.D. of triplicate samples (n = 3). Telomerase activity in each line is expressed as a percent of HeLa cells’ activity.

A panel of 10 pancreatic cancer cell lines was characterized for telomere length and relative telomerase activity. Genomic DNA isolated from each line was digested with a cocktail of 4 bp cutters, resolved on agarose gel and subjected to *in situ* hybridization to a [^32^P]-labeled (TTAGGG)_4_ probe. Signals revealed a smear of telomeric fragments, ranging in size from 1 to 7 kbp (Figure 1A). Densitometric analysis allowed determination of the median size of the telomeric signal for each cell line (Figure 1B). AsPC1 had the shortest telomeres (median = 2.2 kb) whereas CAPAN2 and Panc1 had the longest (medians = 3.5 and 3.7 kb, respectively). In some of the lines, in particular CAPAN1 and CFPAC1, telomere distribution was biphasic. In these lines, an abundance of short telomeres coexisted with a small fraction of much longer telomeres (>5 kb). Expect for CAPAN2 and Panc1, all the lines had very short bulk telomeres, with median sizes ranging from 2.2 to 2.8 kb. As a comparison, telomeres in the normal human pancreas are between 9 and 15 kb in length, depending on the age of the donors [45].

Using a non-radioactive TRAP assay (Telomeric Repeat Amplification Protocol), baseline telomerase activity was measured quantitatively in each cell line. The TRAP assay uses PCR to amplify the products of telomerase elongation (Telomerase ladder) along with an internal telomerase assay standard (ITAS). Telomerase activity is quantified as the ratio of the intensity of the telomerase ladder over that of the ITAS. Figure 1C shows a representative example of a TRAP assay performed on the panel, using the same number of cells from each line. Large differences in the relative intensity of the ITAS and telomerase ladder were observed, indicative of large differences in baseline telomerase activities. To obtain more precise measurements of telomerase activity, serially diluted samples of each line were simultaneously assayed and compared to HeLa cells. Densitometric analysis allowed calculation of baseline telomerase activity for each line (Figure 1D). Pancreatic cancer cell lines had levels of telomerase activity that ranged from 0.5% (CD18) to 23% (AsPC1) of that of HeLa cells. Four lines (L3.6pl, MiaPaCa2, HPAF, AsPC1) were part of a group with higher levels of telomerase (15.8±4.8 percent of HeLa). Six lines (CD18, Panc1, Hs766T, CFPAC1, CAPAN1, CAPAN2) were part of group displaying 10-fold lower levels of telomerase (1.53±1.1% percent of HeLa). A 46-fold difference in telomerase activity was noted between the AsPC1 (highest activity) and CD18 (lowest activity) cells. No correlation was found between the size of telomeres and level of telomerase activity (Figure S1A).

### Telomerase Inhibitory Activity of GRN163L in Pancreatic Cancer Cell Lines

**Figure 2:**
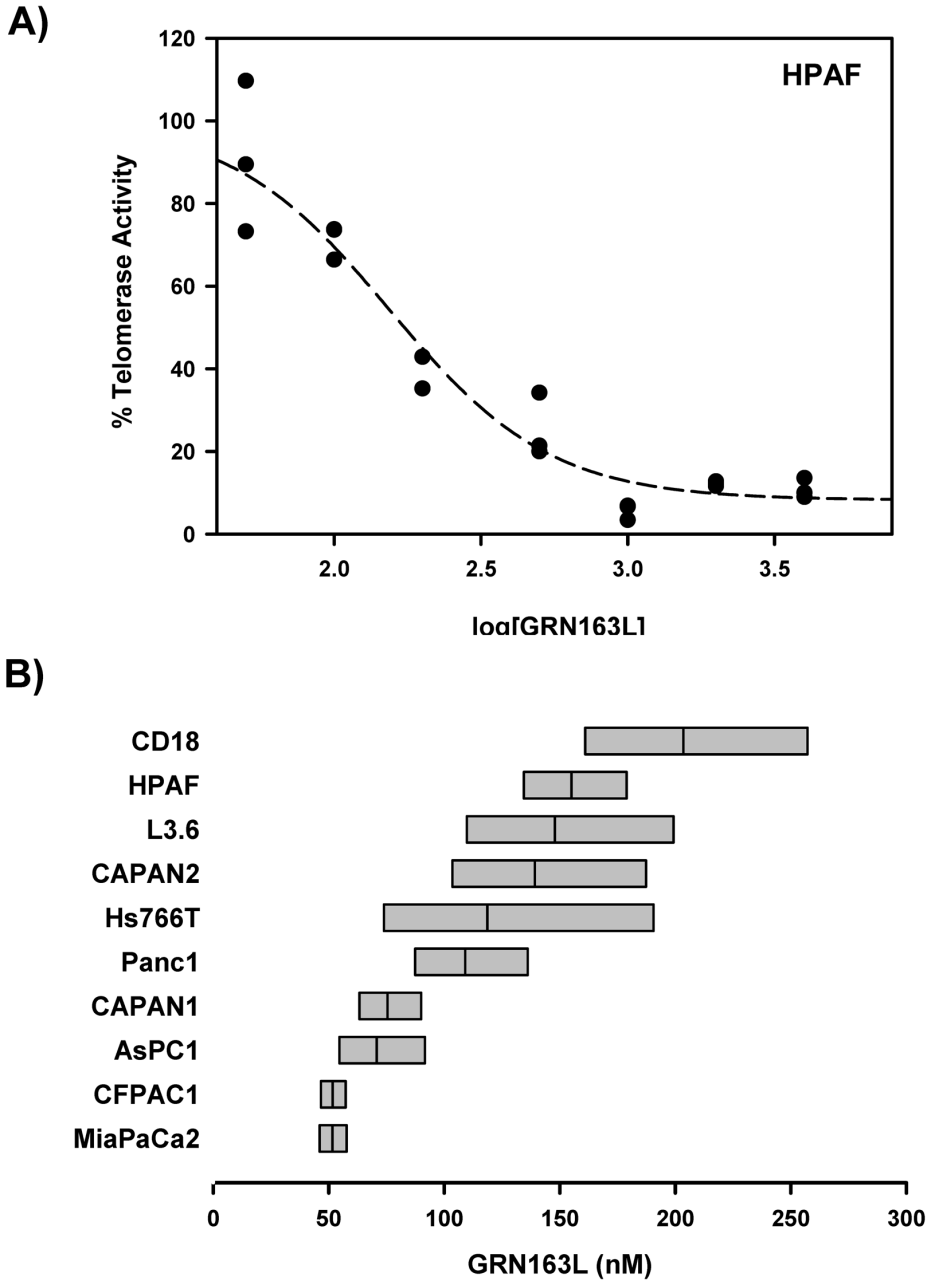
Short-term responses of pancreatic cancer cell lines to GRN163L. A) Dose-Response curve of telomerase inhibition by GRN163L. HPAF cells were treated in triplicate with increasing concentrations of GRN163L (50 nM to 4 µM). Twenty-four hours later, telomerase activity was measured using the TRAP assay. Samples treated with no drug were set to 100%. B) IC_50_ of each line for the inhibition of telomerase by GRN163L. Dose-response curves were performed as in panel A. Non-linear curve fitting allowed calculation of an IC_50_ for each line. Results are expressed as the IC_50_ (middle bar) and its 95% interval of confidence (flanking bars).

Next, we examined the telomerase inhibitory effects of GRN163L in each of the 10 pancreatic cancer cell lines. In each line, telomerase activity was measured at 24 hours following the addition of increasing concentrations of GRN163L to the cells (n = 3 for each dose). Figure 2A shows the results of this analysis in HPAF cells. Densitometric analysis of the TRAP gel allowed measurements of relative telomerase activity, which could then be expressed as a function of GRN163L concentration. These dose-response curves were fitted by nonlinear regression to allow calculation of an IC_50_ for each line. Figure 2B displays the 95% interval of confidence for the value of the IC_50_ in each line. All 10 pancreatic cancer cell lines responded to GRN163L, with IC_50_ that ranged from 50 nM to 200 nM. The least sensitive line was CD18 (IC_50_ = 204 nM) and the most responsive ones were CFPAC1 and MiaPaCa2 (IC_50_ = 52 nM, both). We saw no correlation between baseline telomerase activity and the response of the cell lines to GRN163L, as measured by their respective IC_50_ (Figure S1B).

### Effects of Chronic GRN163L Exposure on Proliferation and Cellular Lifespan

**Figure 3:**
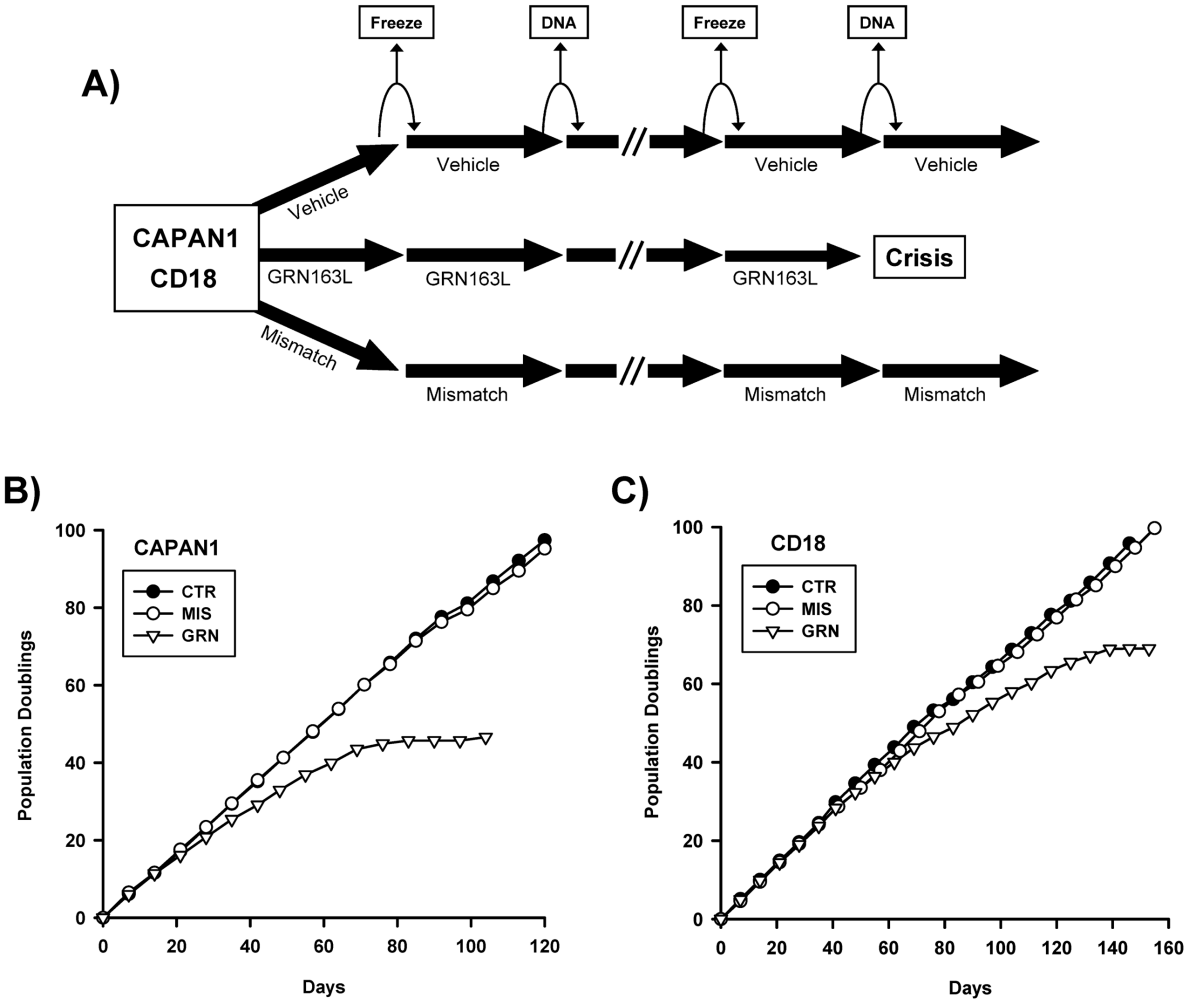
Effects of chronic GRN163L on cellular lifespan. A) Pancreatic cancer cell lines CAPAN1 and CD18 were selected for these studies. Every 2–3 days, each lines was given either no drug (vehicle), GRN163L (1 µM) or the Mismatched oligo (1 µM). Once a week, cells were counted and the results are plotted as the number of population doublings achieved as a function of time. Every other week, samples were put aside for TRF analysis (DNA) or frozen down as backup. B,C) Growth curves of the drug-treated CAPAN1 (B) and CD18 (C) cells are shown.

Two pancreatic cancer cell lines were chosen to study the effects of long-term exposure to GRN163L: CAPAN1 and CD18. Comparable to the majority of the lines surveyed, CAPAN1 and CD18 both had relatively short telomeres (2–3 kb) and low level telomerase activities (0.5–1% of HeLa cells). However, the two lines differed in their response to GRN163L, with the CAPAN1 cells exhibiting a higher sensitivity (IC_50_ = 75 nM) to GRN163L than CD18 cells (IC_50_ = 204 nM). To measure the effects of continuous GRN163L exposure, the two cell lines maintained in continuous log phase growth in the presence of no drug (CTR), 1 µM GRN163L (GRN), or 1 µM of a mismatched control oligo (MIS). This mismatched oligo had the same modified chemistry as GRN163L (palmitate-conjugated thio-phosphoramidate) but with mismatched bases to block binding to the hTR template (5′-palmitate-TAGGTGTAAGCAA-NH_2_-3′; mismatched positions underlined). Drugs were replaced every 2–3 days and once a week, cells were counted and re-seeded at lower densities, with the excess cells saved for either DNA isolation or for making frozen stocks (Figure 3A).

**Figure 4:**
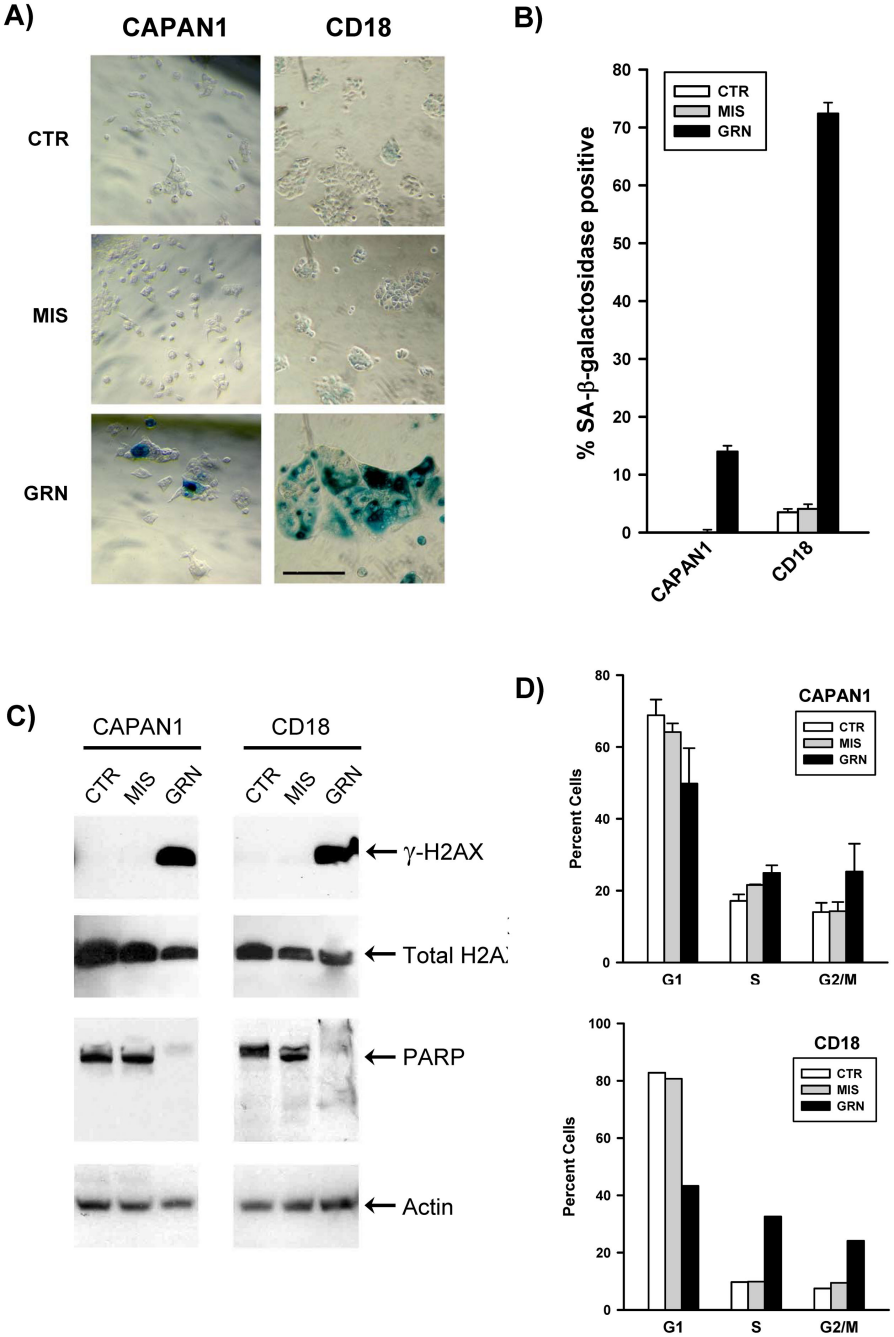
Markers of apoptosis, senescence and DNA damage response in the GRN163L-treated CAPAN1 and CD18 cells. At the end of the growth curves presented in Figure 3, cells treated with no drug (CTR), mismatch oligo (MIS) and GRN163L (GRN) were analyzed for SA-β-galactosidase activity. A) Histological analysis of SA-β-galactosidase activity. Histochemical staining reveals SA-β-galactosidase activity in the GRN163L-treated CAPAN1 and CD18 cells, as evidenced by the deposition of insoluble blue pigments (Right panels). B) Percent of cells in each sample that stained positive for SA-β-galactosidase activity. Measurements were done in triplicates (Mean ± S.D., n = 3). C) Western blot analysis. Samples were probed with antibodies against histone H2AX, phosphorylated H2AX (γ-H2AX), PARP and actin. D) Flow cytometric analysis of DNA content. Cells were stained with propidium iodide and analyzed for DNA content. Measurements were made twice at one week interval for the CAPAN1 (Mean ± S.D., n = 2). In the case of the CD18, one measurement only could be made before the GRN163L-treated cells were lost to crisis (n = 1).

Figures 3B and 3C display the effects of long-term GRN163L exposure on the proliferation and lifespan of the two cell lines. In both lines, control populations treated with no drug (CTR) or with the mismatched oligo (MIS) grew at relatively constant rates throughout to produce curves that were almost identical. In their first 3–8 weeks of growth, the GRN163L-treated cells (GRN) grew as fast as their corresponding control populations. But thereafter, proliferation of these cells declined progressively as they began to experience crisis. Crisis in these cultures was characterized by the appearance of senescent cells (Figures 4A and 4B) and by the ever increasing accumulation of floating cells, indicative of cell death (Data not shown). Eventually, weekly cell counts began to yield lower numbers of cells after growth than what had been plated a week prior, thereby giving rise to a stationary phase or plateau. Eventually, loss of the CAPAN1 and CD18 cells respectively occurred after a total of 47 and 69 doublings done in the presence of GRN163L (Figures 3B and 3C).

At the end of the growth curves, cells were examined for markers of senescence and apoptosis (Figure 4). The GRN163L-treated populations contained adherent cells exhibiting the flat and enlarged phenotype associated with senescence (Figure 4A). Staining for SA-β-galactosidase activity, a marker of senescence, confirmed that these enlarged cells were experiencing senescence (Figures 4A and 4B). A significant fraction of cells expressing the activity was observed in the GRN163L-treated CAPAN1 and CD18 cells but not in control populations (CTR, MIS). At the end of the growth curves, cells were subjected to cell cycle analysis, using flow cytometry to measure DNA content. Compared to their corresponding controls (CTR, MIS), the GRN163L-treated populations (GRN) consistently exhibited a higher fraction of cells with an S or G2/M DNA content and lower percent of cells in the G1 phase (Figure 4D). Also noted in the GRN163L-treated samples were large increases in cells containing a sub-G1 DNA content indicative of apoptosis (Figure S2) [46]. To confirm the induction of apoptosis, samples were Western blotted for levels of full-length PARP1 (Poly(ADP-Ribose) Polymerase 1). PARP1 is cleaved by Caspase-3 during the executive phase of apoptosis, and this cleavage is a hallmark of apoptosis [47]. As shown in Figure 4C, PARP1 was nearly completely cleaved in the GRN163L-treated CAPAN1 and CD18 cells as compared to their corresponding controls (CTR, MIS). We also have examined the cells for evidence of an activated DNA damage response, using phosphorylated H2AX (γ-H2AX) as a marker [48]. As shown in Figure 4C, H2AX was phosphorylated in the GRN163L-treated cells (GRN) but not in their corresponding controls (CTR, MIS). Collectively, these results indicate that long-term exposure of the CAPAN1 and CD18 cells to GRN163L leads to the induction of a crisis characterized by senescence, apoptosis and DNA damage response.

### Effects of Chronic GRN163L Exposure on Telomere Maintenance

**Figure 5:**
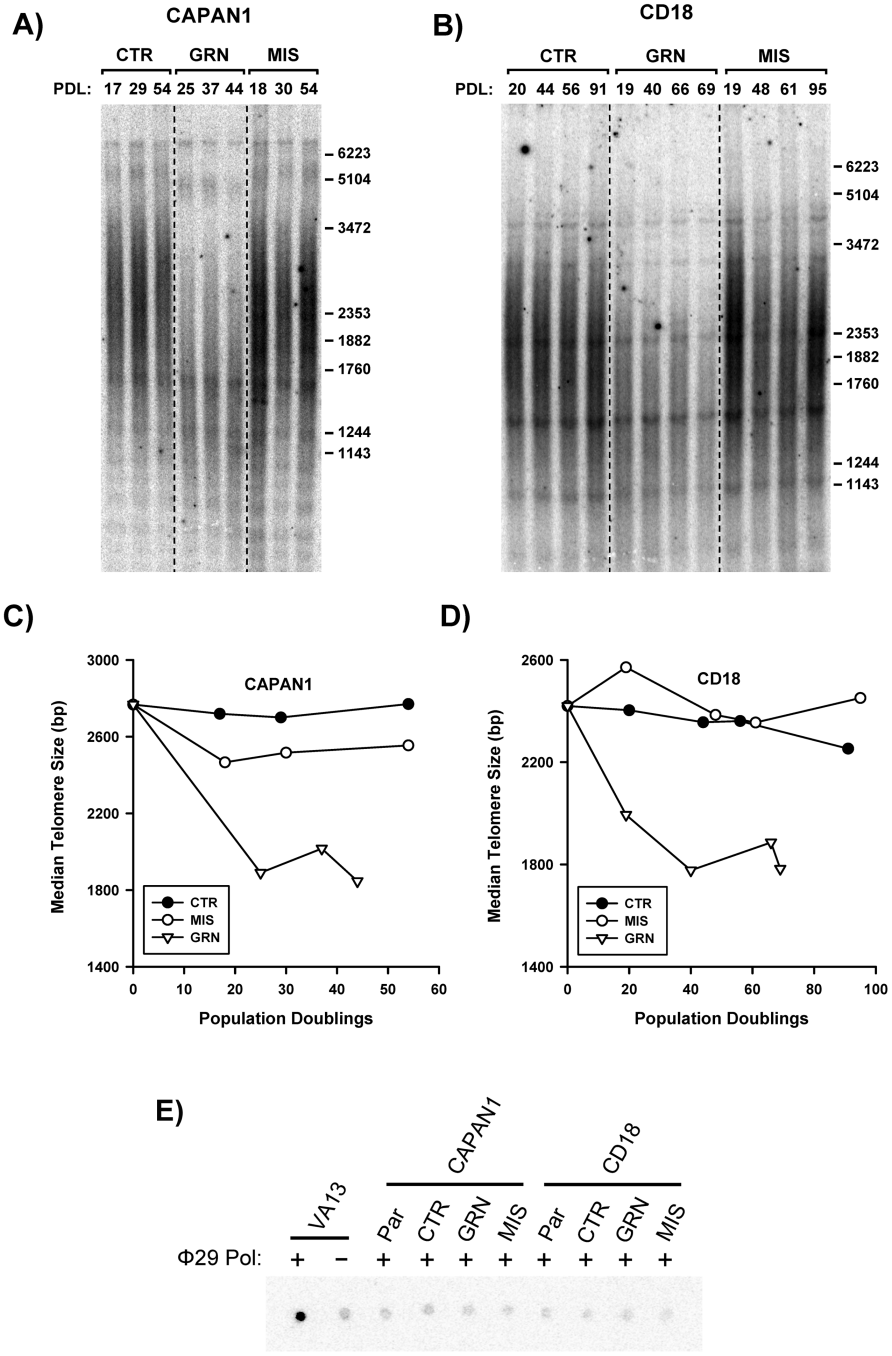
Effects of chronic GRN163L on telomere maintenance. Cells were treated every 2–3 days with no drug (CTR), mismatch oligo (MIS) or GRN163L (GRN). A–B) Southern blot analysis of the telomeres. At the indicated population doubling (PD), genomic DNA samples were collected and subsequently processed for telomere size measurements, as described in Figure 1A. C–D) Telomere size measurements. Median telomere sizes are shown for the drug- and control-treated CAPAN1 and CD18 cells. E) Detection of ALT by quantitative PCR. Samples tested included CAPAN1 and CD18 cells treated with no drug (CTR), mismatch oligo (MIS) and GRN163L (GRN), all of which harvested at the end of the growth curves presented in Figure 3. Also included were stocks of CAPAN1, CD18 and VA13 cells. Samples were tested in triplicate with (+) and without (−) the Φ29 DNA polymerase. A representative dot blot is shown.

Figure 5 describes the effects of continuous GRN163L on the maintenance of telomeres. In the control populations (CTR, MIS), telomere sizes were relatively stable over time in both of the cell lines. In the GRN163L-treated cells, telomeres had already become shortened by the time they were first analyzed (PD 25 for CAPAN1; PD 19 for CD18). At this first time point, telomere sizes had already declined to a median size of less than 2.0 kb (Figures 5C, 5D). In the GRN163L-treated CAPAN1 cells, this first time point coincided with the start of crisis, when cells began to experience reduced proliferation (Figure S3). However, for the remaining time points and throughout crisis, telomeres in these CAPAN1 cells remained short but stable (Figures 5A, 5C). In the GRN163L-treated CD18 cells, additional shortening took place after the first time point, with telomeres reaching their minimum size at PD 40, when cells began to experience crisis (Figure S3). But thereafter and throughout crisis, telomeres in these CD18 cells remained short but stable (Figures 5B, 5D). At the last time point, just before the two GRN163L-treated cell lines were lost to crisis, telomeres were still in the same 1.8 to 2.0 kb range.

**Figure 6:**
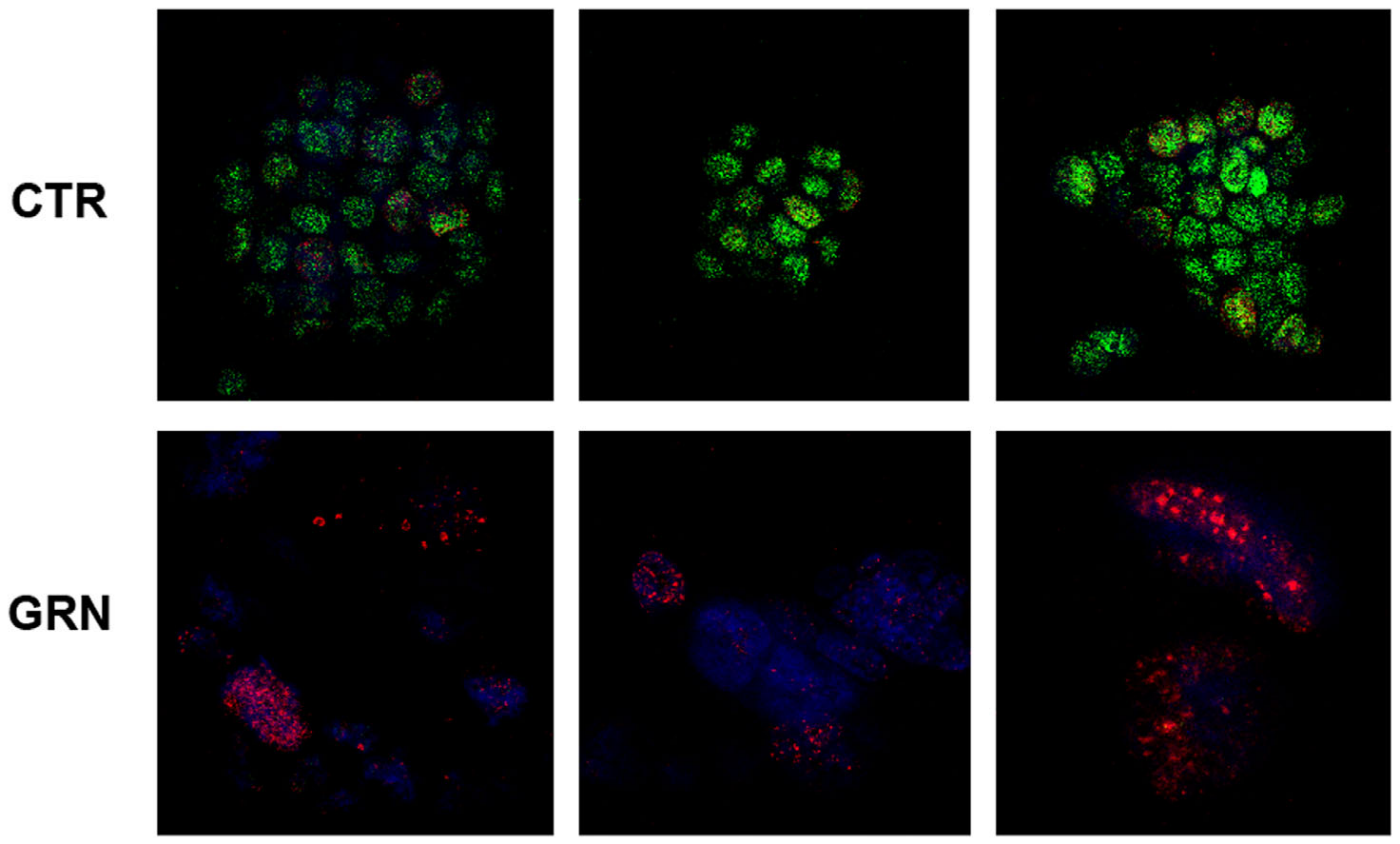
Immunofluorescence analysis of telomeres in the GRN163L-treated CD18 cells. CD18 treated with GRN163L (GRN) or with no drug (CTR) were harvested at the end of the growth curve presented in Figure 3. Cells were fixed and stained with antibodies against TRF2 (green) and γ-H2AX (red) and were counter-stained with DAPI (blue). Images were visualized on a confocal microscope (Zeiss 510 Meta Confocal Laser Scanning Microscope). Three representative images of each sample are shown.

CD18 samples harvested at the end of the growth curve (Figure 3C) were subjected to immunofluorescence analysis of their telomeres (Figure 6). CD18 treated with GRN163L (GRN) or with no drug (CTR) were stained with antibodies against γ-H2AX (Cy5, red) and the telomere-associated protein TRF2 (FITC, green). In the control sample, confocal microscopy revealed an abundance of punctate signals for TRF2 in the nuclei, which corresponded to individual telomeres. Less than 20% of these nuclei also contained punctate signals for γ-H2AX (16±4%; n = 6). In the GRN163L-treated samples, the opposite results were obtained. Only a few telomeres carried sufficient telomeric repeats to allow detection by the anti-TRF2 antibody. In addition, a majority of these nuclei displayed an abundance of γ-H2AX foci (>10 foci/nucleus), indicative of a ds-DNA break (83±30%; n = 6). These results show that in the GRN163L-treated cells, telomeres have become uniformly short to the point of undetectability. They also show evidence of widespread DNA damage characteristic of crisis in approximately half the cells.

**Figure 7:**
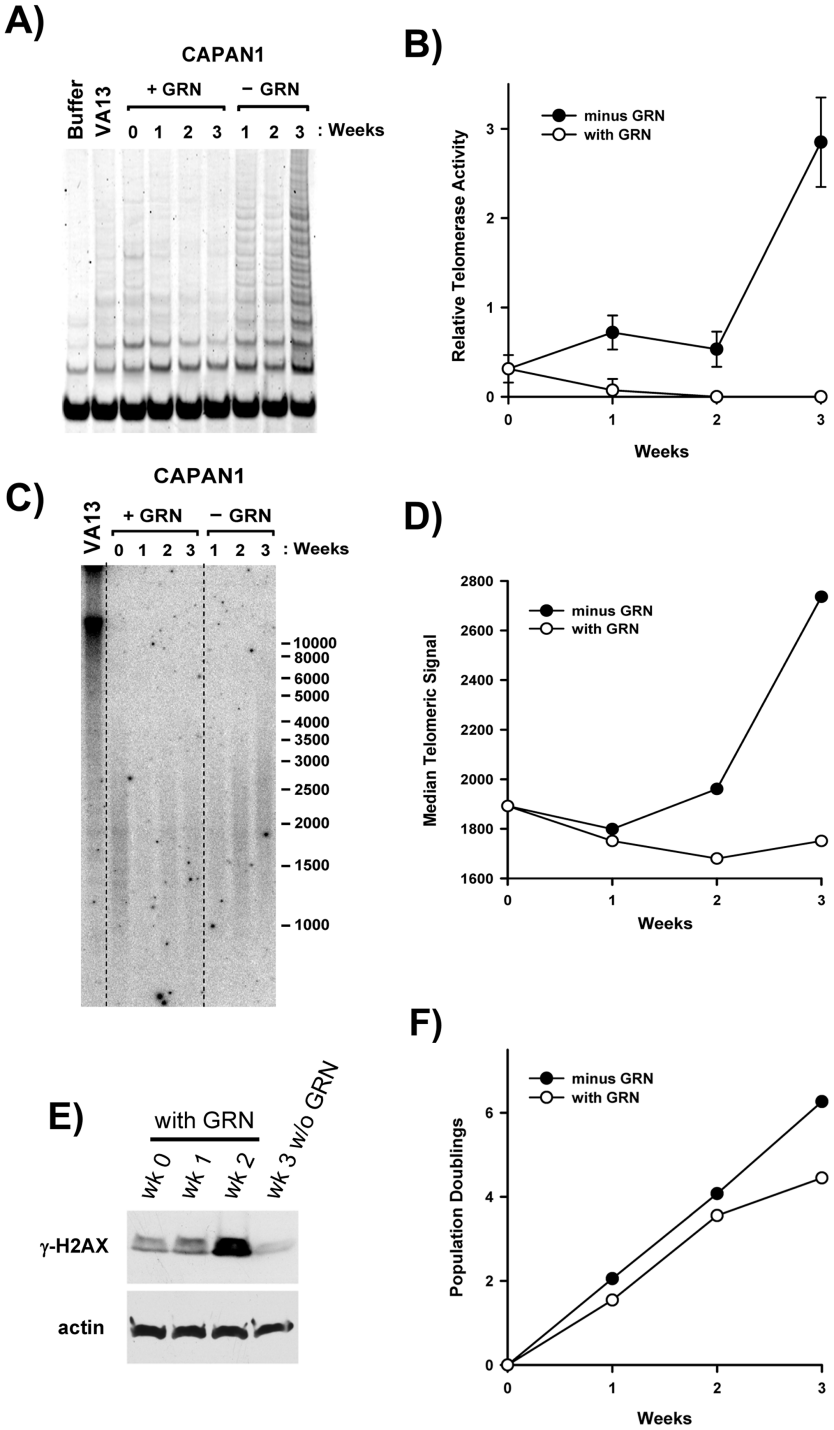
Recovery from chronic GRN163L exposure after removal of the drug. After 63 days of continuous GRN163L exposure, CAPAN1 cells were sub-cultivated for 3 weeks in the presence or absence of GRN163L. A, B) Quantification of telomerase activity. Telomerase activity was measured using the TRAP assay at time 0 and after 1, 2, and 3 weeks. Relative telomerase activity was calculated as the ratio of the intensity of the telomerase ladder over the intensity of the ITAS. Each measurement is the Mean ± S.D. of triplicate samples (n = 3). C, D) Telomere size measurements. Telomeres were analyzed at time 0 and after 1, 2, and 3 weeks. Median telomere size was estimated as the size corresponding to half the cumulative sum of the intensities. E) Levels of the γ-H2AX. The indicated samples were Western blotted with antibodies against phosphorylated H2AX (γ-H2AX) and actin. F) Growth rate measurements. Once a week, cells were counted and the number of population doublings done was plotted as a function of time.

In human cancer cells, telomeres are maintained by two possible mechanisms: telomerase and the alternative lengthening of telomeres (ALT). The ALT mechanism is characterized by long and heterogeneous telomeres (e.g. VA13 cells, Figure 7C) and by an abundance of telomeric C-rich circles [49], [50]. To address the possibility that the stabilization of telomeres observed in the GRN163L-treated cells might be driven by ALT, cells were assayed for the presence of telomeric C-rich circles. In this assay, the circles serve as self-priming DNA template for rolling circle amplification by the Φ29 DNA polymerase, producing long G-rich telomeric DNA molecules which can then be detected by dot blot using a [^32^P]-(TAACCC)_4_ probe [44]. The VA13 cell line, which uses ALT to maintain telomeres [41], was used as positive control. As Figure 5E shows, incubation of VA13 DNA with the Φ29 DNA polymerase led to an abundant production of G-rich telomeric DNA, indicative of ALT. This abundant production of G-rich telomeric DNA was not detected in polymerase reactions driven by DNA isolated from the GRN163L-treated CAPAN1 or CD18 cells (Figure 5E). These results indicate that the ALT mechanism of telomere maintenance is not implicated in the stabilization of telomeres observed in the GRN163L-treated pancreatic cancer cells.

### Telomere Recovery After GRN163L Removal

To exclude the possibility that the stabilization of telomeres observed in the GRN163L-treated cells may have occurred as a consequence of insufficient GRN163L exposure, we examined the time course of telomere recovery after the removal of GRN163L. At PD 40 (Day 62 from Figure 3B), GRN163L-treated CAPAN1 cells were switched to GRN163L-free culture conditions. For three weeks, the cells were cultivated in either of the absence or presence of GRN163L. Cultivation in the absence of GRN163L led to a gradual reactivation of telomerase (Figures 7A, 7B). At 1 and 2 weeks after drug removal, a partial reactivation of telomerase was observed. This was followed by a much larger increase in telomerase activity detected in the third week without GRN163L. The recovery of telomeres followed a similar time course (Figures 7C, 7D). No significant re-elongation of telomeres was observed until after 3 weeks of growth in the absence of the drug. In the third week, telomeres in the GRN163L-free samples grew at a rate of ∼100 bp/day (Figure 7D). This telomere recovery was accompanied by a dramatic reduction in the level of γ-H2AX (Figure 7E). In the GRN163L-treated samples, γ-H2AX was gradually induced as cells approached the end of their lifespan. By the second week of the experiment (or after 76 days of continuous GRN163L exposure), the marker was already maximally induced. But after three weeks of growth in the absence of GRN163L, γ-H2AX had already decreased to below its level detected at time 0 (wk 0, equal to 62 days of continuous treatment). In the third week, differences in cell proliferation could also be observed between the GRN163L-treated and -untreated populations (Figure 7F). In the third week, the GRN163L-treated cells exhibited a sharp decline in growth rate, whereas those switched to the GRN163L-free conditions showed no decline in proliferation. These results indicate that the effects of GRN163L are reversible, but that recovery from chronic exposure to the drug takes three weeks. Our thrice weekly doses of GRN163L should therefore have constituted an adequate schedule for constant telomerase inhibition.

## Discussion

Telomere shortening is the earliest and most common genetic alteration acquired during pancreatic cancer development. This alteration, detected in 96% of PanIN precursor lesions, is accompanied by evidence of a DNA damage response consistent with telomere uncapping and dysfunction [51], [52]. Not surprisingly, more than 90% of these tumors eventually re-activate telomerase, which otherwise remains undetectable in normal pancreatic tissues. Yet, in spite of active telomerase, the majority of advanced pancreatic tumors harbor short telomeres and chromosomal ends that lack detectable telomeric repeats. In agreement with these findings, we have found that the great majority of pancreatic cancer cell lines (8/10 lines) have very short telomeres, in the range of 2 to 3 kb only (Figure 1B). These very short telomeres were expected to make these cells particularly susceptible to the effects of chronic telomerase inhibition. We also have surveyed the panel for levels of telomerase activity, and an unexpected finding was the large differences in activities between the cell lines (Figure 1D). The cell lines belonged to one of two groups, exhibiting either a higher (L3.6pl, MiaPaCa2, HPAF, AsPC1) or 10-fold lower level of activity (CD18, Panc1, Hs766T, CFPAC1, CAPAN1, CAPAN2), with the highest (AsPC1) and lowest (CD18) expressers exhibiting a 46-fold difference in activity. The significance of these large differences between the lines is unclear, as telomerase activity did not correlate with either telomere size or response to GRN163L (Figure S1). The lack of correlation with telomere size was no surprise since telomere length is regulated not just by the activity of telomerase but even more significantly by the Shelterin complex [8]. All ten lines of the panel responded well to the telomerase inhibitory activity GRN163L (Figure 2B). With IC_50_ in the nanomolar range, the response of pancreatic cancer cell lines to the drug was comparable if not better than previously reported for other cancer cell lines [20], [35].

Using CAPAN1 and CD18 as models, we examined the effects of continuous telomerase inhibition on pancreatic cancer cells. As expected for a telomerase inhibitor, GRN163L affected proliferation and survival only after a delay during which sufficient telomere shortening needed to occur (Figures 3 and 5). This telomere shortening was elicited by GRN163L but not by the mismatch oligo (Figure 5), which possesses the same lipid-conjugated thio-phosphoramidate chemistry as GRN163L but is unable to hybridize to hTR. At the end of the lifespan of the GRN163L-treated CD18 cells, telomeres had become critically short to the point of undetectability (Figure 6). Only a few telomeres carried sufficient telomeric repeats to allow detection by the anti-TRF2 antibody. Southern blot analysis suggested that these telomeres still carried 1.8 kb of repeats (Figure 5D). However, this method overestimates the length of telomeres. Recalculating median telomere size after correcting for signal intensity, as we have done before [53], returned a value of just 1.1 kb, with some of that still representing sub-telomeric DNA (data not shown). Most importantly, more than 80% of the GRN163L-treated cells also displayed an abundance of γ-H2AX foci indicative of the presence of ds-DNA-breaks (Figure 6), as expected for cells in crisis after cycles of telomere fusion, anaphase-bridge, and breakage. Western blot analysis also indicated a strong induction of γ-H2AX in the GRN163L-treated cells but not in the corresponding controls (Figure 4C). This induction of γ-H2AX was highest at the end of lifespan, but could already be detected 3 weeks prior to the loss of the cultures (Figure 7E). Taken together, these results are consistent with GRN163L limiting lifespan by the gradual shortening and uncapping of telomeres. This interpretation is strengthened by the observation that the removal of GRN163L reverses most of these effects, allowing a reactivation of telomerase, re-elongation of telomeres, extinction of γ-H2AX induction, and escape from crisis.

An important finding was the biphasic response of telomeres to GRN163L. In both CAPAN1 and CD18 cells, almost all of the shortening took place within the first few weeks of exposure to GRN163L (Figure 5). However, as soon as the cells began to experience reduced proliferation (Figure S3), telomeres became stable and showed no additional changes in signal intensity (Figures 5A, 5B), median size (Figures 5C, 5D, and S3) or even size distribution (Figure S4). This stabilization was not a consequence of the activation of ALT (Figures 5E, 7C) nor was it due to inadequate drug scheduling or development of GRN163L resistance, as removal of the drug led to a gradual re-elongation of the telomeres (Figure 7). Importantly, a similar stabilization of telomeres has also been observed in cancer cells treated with the small telomerase inhibitor MST-132 [54], [55]. We also have reported a similar telomere stabilization in hTERT-immortalized cells expressing limiting amount of telomerase [53]. Under conditions of limiting telomerase activity, the longest telomeres shorten but the size of the shortest telomeres is maintained. The net result is the accumulation of cells that continue to proliferate with exceptionally short but functional telomeres [53]. This stabilization and accumulation of extra short telomeres is though to be the product of cis-acting regulatory mechanisms that preferentially recruit telomerase to elongate the shortest telomeres. In humans, this regulation is exerted by the Shelterin complex, which binds simultaneously to duplex telomeric DNA and the telomeric 3′-overhang, the substrate of telomerase [56]. The complex regulates telomere length by inhibiting the access of telomerase to the overhang [57], [58]. The stabilization of telomeres in the GRN163L-treated CD18 and CAPAN1 cells is likely to be the product of this regulatory function exerted by the Shelterin complex. This hypothesis is supported by the presence in the GRN163L-treated CD18 population of a small subset of cells (17%) with extremely short (no TRF2 signal) but functional telomeres (no γ-H2AX foci) (Figure 7). Once telomeres have become critically shortened, the Shelterin complex may no longer have been able to block telomerase, thereby making these telomeres an exceptionally good substrate for residual traces of telomerase activity. Overcoming this pitfall will require inhibitors that more completely inhibit telomerase and/or drugs that boost the activity of the Shelterin complex. In support of this concept, MST-132 has been reported to synergize with Tankyrase inhibitors to shorten telomeres faster and induce crisis earlier [54], [55]. Tankyrases are poly(ADP-ribose)polymerases that parsylate TRF1, and their inhibition increases the telomerase-inhibitory activity of the Shelterin complex [54], [59], [60].

According to the two stage model of human cell mortality, telomere shortening can result in the induction of either senescence or crisis, depending on the functionality of the p53 and p16/pRB pathways [4], [11]. The two pathways are key components of the machinery that triggers senescence in response to telomere dysfunction. Because p53 and p16 are both inactivated in CAPAN1 cells [61], we expected these cells to have a reduced propensity to senesce. Consistent with this prediction, only 14% of GRN163L-treated CAPAN1 cells were experiencing senescence at the end of their lifespan (Figure 4B). In CAPAN1 cells, the predominant response to terminal telomere shortening was crisis, as evidenced by the presence of markers of programmed cell death (floating dead cells, PARP1 cleavage, and sub-G1 DNA content). In CD18 cells, p53 is mutated but cells express the wild-type p16 protein gene [61]. Perhaps because of the functionality of p16, we saw a much greater proportion (∼ 74%) of GRN163L-treated CD18 cells experiencing senescence (Figure 4B). But if senescence in normal cells is a quiescent but viable state [4], [11], senescence in the GRN163L-treated CD18 cells appears to be associated with a loss of viability, as evidenced by the ever increasing number of floating dead cells and eventual loss of the culture, as well as markers of apoptosis (sub-G1 DNA content, PARP1 cleavage). It may be that telomere dysfunction is inducing senescence first, but as cells eventually overcome the p16-mediated G1/S checkpoint, fused telomeres are able to make it through anaphase where they cause mitotic catastrophe and apoptosis. In support of this sequential induction of senescence then crisis, we discovered that most floating dead cells were still positive for the activity of SA-β-galactosidase, even if some of the activity had leached out the dead cells (Figure S5). We also noted an accumulation of cells with a 4 N DNA content, as one would expect if uncapped telomeres and telomere fusions are respectively allowed to reach the G2/M checkpoint and metaphase/anaphase transition (Figure 4D). If senescence in normal cells is a quiescent state that maintains viability [11], senescence in CD18 cells does not appear to provide protection against cell death. For cancer therapy, this ultimate demise of the senescent cells has the advantage of excluding the possibility that these cells might on day regain the ability to proliferate.

An important finding with implications in the design of GRN163L-based therapies was the slow time course of recovery after the removal of the drug (Figure 7). Only in the third week following GRN163L removal did we observe substantial telomerase reactivation and telomere re-elongation. This persistence of the effects of GRN163L is potentially made possible by the stability of the drug [62], irreversibility of the inhibition [62], and slow turnover of the telomerase complex [63]. Telomerase was also reported to be less processive in the first few weeks following the reversal of long-term exposure to GRN163L, as detected by measurements of native telomere extension by telomerase [64]. This loss of processivity correlated with a failure of Cajal bodies to deliver telomerase to telomeres in the first weeks following the removal of the drug. Once telomerase is inhibited in a patient’s tumor, a maintenance dose given once every other week might therefore be sufficient to maintain continuous telomerase inhibition, thereby reducing the risk of side effects.

Pancreatic cancer has one of the highest rates of recurrence following surgical resection, the only curative treatment for the disease [3]. In the resectable population, telomerase inhibitors could potentially be valuable to block the regrowth of residual disease and prevent recurrences. In this report, we demonstrate that the immortal phenotype of pancreatic cancer cells can be reversed by continuous exposure to GRN163L. However, a potential pitfall that could limit the clinical value of GRN163L in pancreatic cancer will be the stabilization of telomeres seen after the initial rapid shortening and the long delays incurred before cells succumb to crisis. Our laboratory is currently investigating the role of the Shelterin complex in mediating these effects. Tankyrase inhibitors are also being tested for their ability to synergize with GRN163L.

## Supporting Information

Figure S1 **Lack of correlation among pancreatic cancer cell lines between levels of telomerase activity and size of telomeres and response to GRN163L.** A) Median size of telomeres of each of the 10 pancreatic cancer cell lines as a function of their respective level of telomerase activity. B) Responses to GRN163L of each of the 10 cell lines as a function of their respective level of telomerase activity.
(TIF)

Figure S2 **Percent of GRN163L-treated cells with a sub-G1 DNA content.** At the end of the growth curves, GRN163L-treated (GRN) and control (CTR, MIS) populations were analyzed for DNA content. Adherent cells were collected by trypsinization followed by centrifugation. Floating cells were collected by centrifugation, and the combined adherent and floating pool was analyzed by flow cytometry. Results are expressed as the percent of the total cells with a sub-G1 DNA content.
(TIF)

Figure S3 **Proliferation rates and telomere sizes as a function of chronological time in the GRN163L-treated CAPAN1 and CD18 cells.** The number of population doublings achieved (Red circles) and median sizes of telomeres (Blue circles) are plotted as a function of chronological time (Days). The data shown is for the GRN163L-treated populations of CAPAN1 and CD18 cells. Dotted black lines extrapolate the initial growth rates of the GRN163L-treated populations before the start of crisis.
(TIF)

Figure S4 **Telomere size distribution over time in the GRN163L-treated CAPAN1 and CD18 cells.** Telomere signal intensity was plotted as a function of the estimated telomere size, as described in the Materials and Methods section. Telomere size distributions in the control populations (CTR, MIS) did not change as a function of population doublings. For the sake of simplicity, only the first time point is shown for each of the control populations (CTR, MIS).
(TIF)

Figure S5 **SA-β-galactosidase activity in the floating dead cells harvested from the GRN163L-treated CD18 cells.** At the end of the CD18 growth curve, floating cells were collected from the GRN163L-treated (GRN) and control (CTR, MIS) populations. Cells were resuspended in 10 pellet volumes of medium. Suspended cells were fixed, washed, and stained for SA-β-galactosidase activity. Washes before steps were performed by centrifugation.
(TIF)
