# Supplementary figures and images for: Telomerase Inhibitor Imetelstat (GRN163L) Limits the Lifespan of Human Pancreatic Cancer Cells
Source: PLoS One. 2014 Jan 7;9(1):e85155. doi: 10.1371/journal.pone.0085155 (PMC3883701; doi:10.1371/journal.pone.0085155)

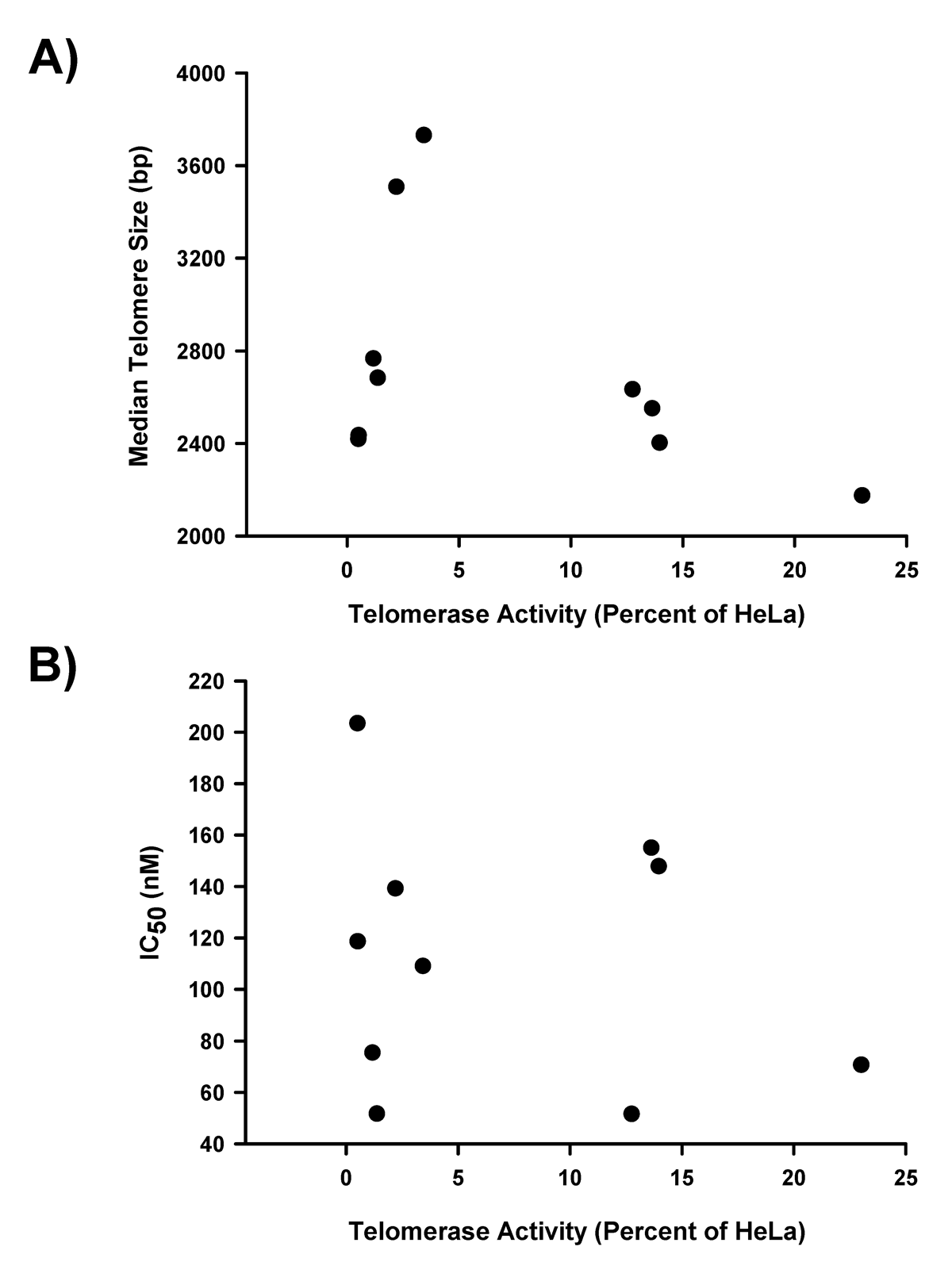

Supplement: Figure S1 — Lack of correlation among pancreatic cancer cell lines between levels of telomerase activity and size of telomeres and response to GRN163L. A) Median size of telomeres of each of the 10 pancreatic cancer cell lines as a function of their respective level of telomerase activity. B) Responses to GRN163L of each of the 10 cell lines as a function of their respective level of telomerase activity. (TIF) [file pone.0085155.s001.tif]

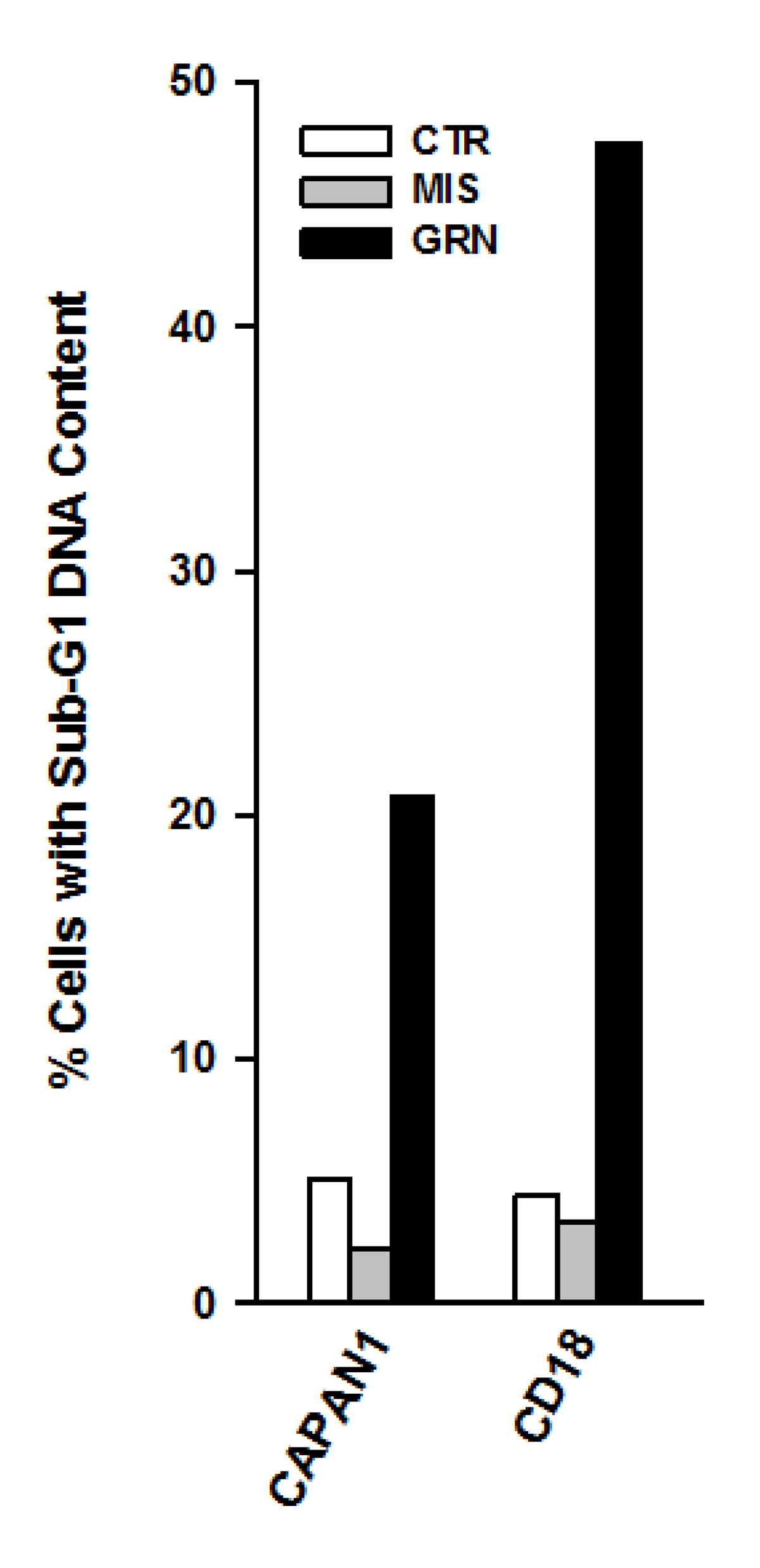

Supplement: Figure S2 — Percent of GRN163L-treated cells with a sub-G1 DNA content. At the end of the growth curves, GRN163L-treated (GRN) and control (CTR, MIS) populations were analyzed for DNA content. Adherent cells were collected by trypsinization followed by centrifugation. Floating cells were collected by centrifugation, and the combined adherent and floating pool was analyzed by flow cytometry. Results are expressed as the percent of the total cells with a sub-G1 DNA content. (TIF) [file pone.0085155.s002.tif]

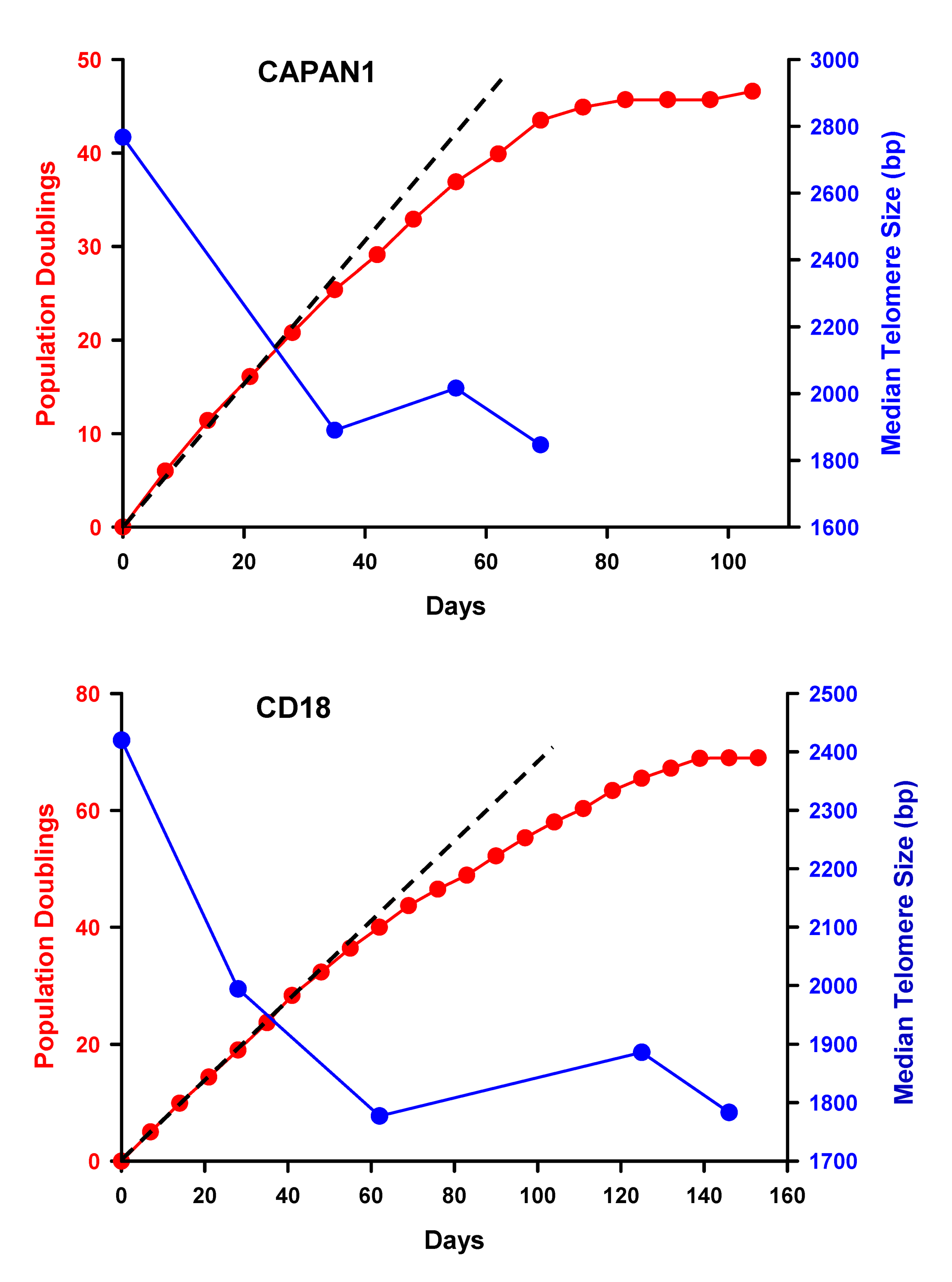

Supplement: Figure S3 — Proliferation rates and telomere sizes as a function of chronological time in the GRN163L-treated CAPAN1 and CD18 cells. The number of population doublings achieved (Red circles) and median sizes of telomeres (Blue circles) are plotted as a function of chronological time (Days). The data shown is for the GRN163L-treated populations of CAPAN1 and CD18 cells. Dotted black lines extrapolate the initial growth rates of the GRN163L-treated populations before the start of crisis. (TIF) [file pone.0085155.s003.tif]

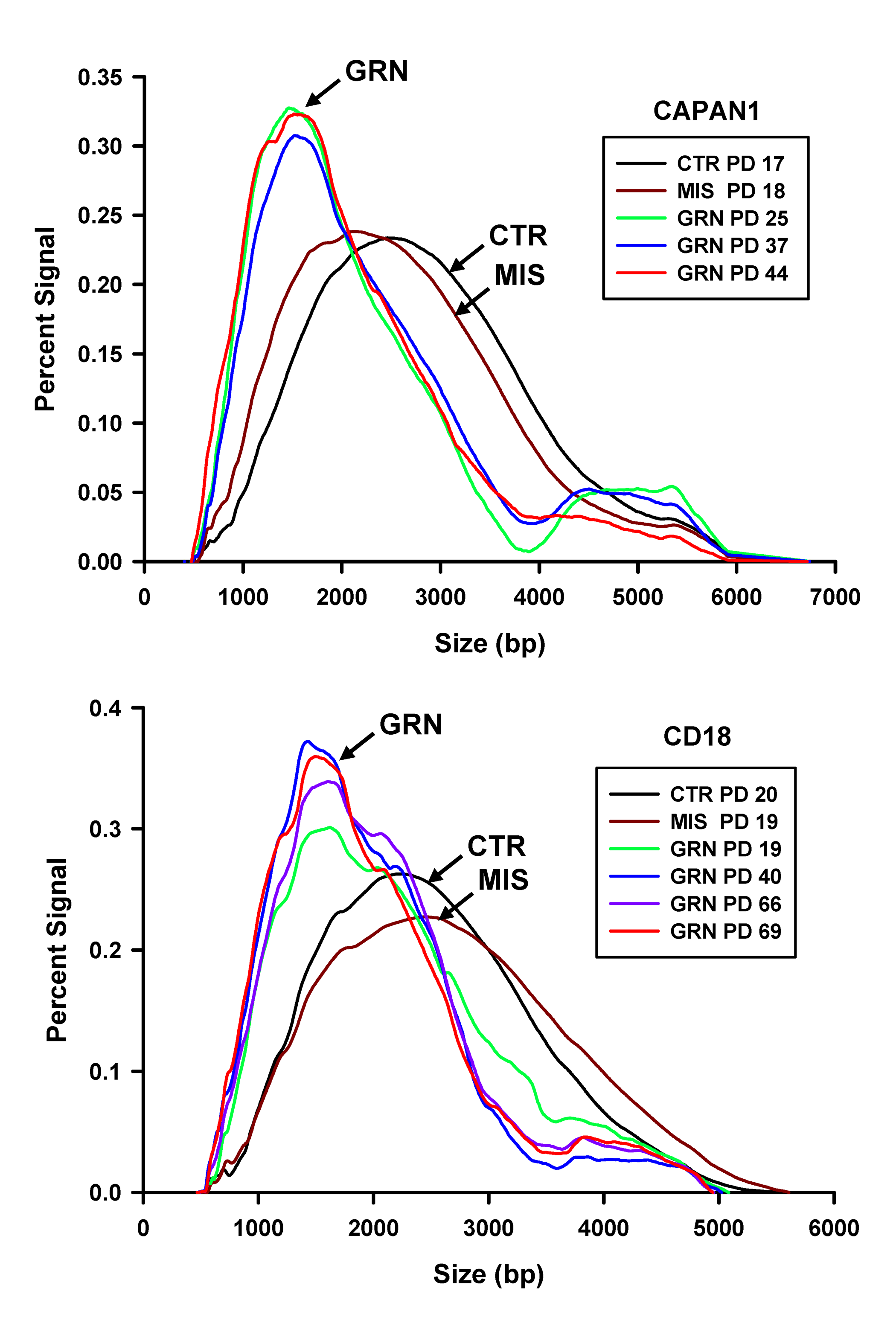

Supplement: Figure S4 — Telomere size distribution over time in the GRN163L-treated CAPAN1 and CD18 cells. Telomere signal intensity was plotted as a function of the estimated telomere size, as described in the Materials and Methods section. Telomere size distributions in the control populations (CTR, MIS) did not change as a function of population doublings. For the sake of simplicity, only the first time point is shown for each of the control populations (CTR, MIS). (TIF) [file pone.0085155.s004.tif]

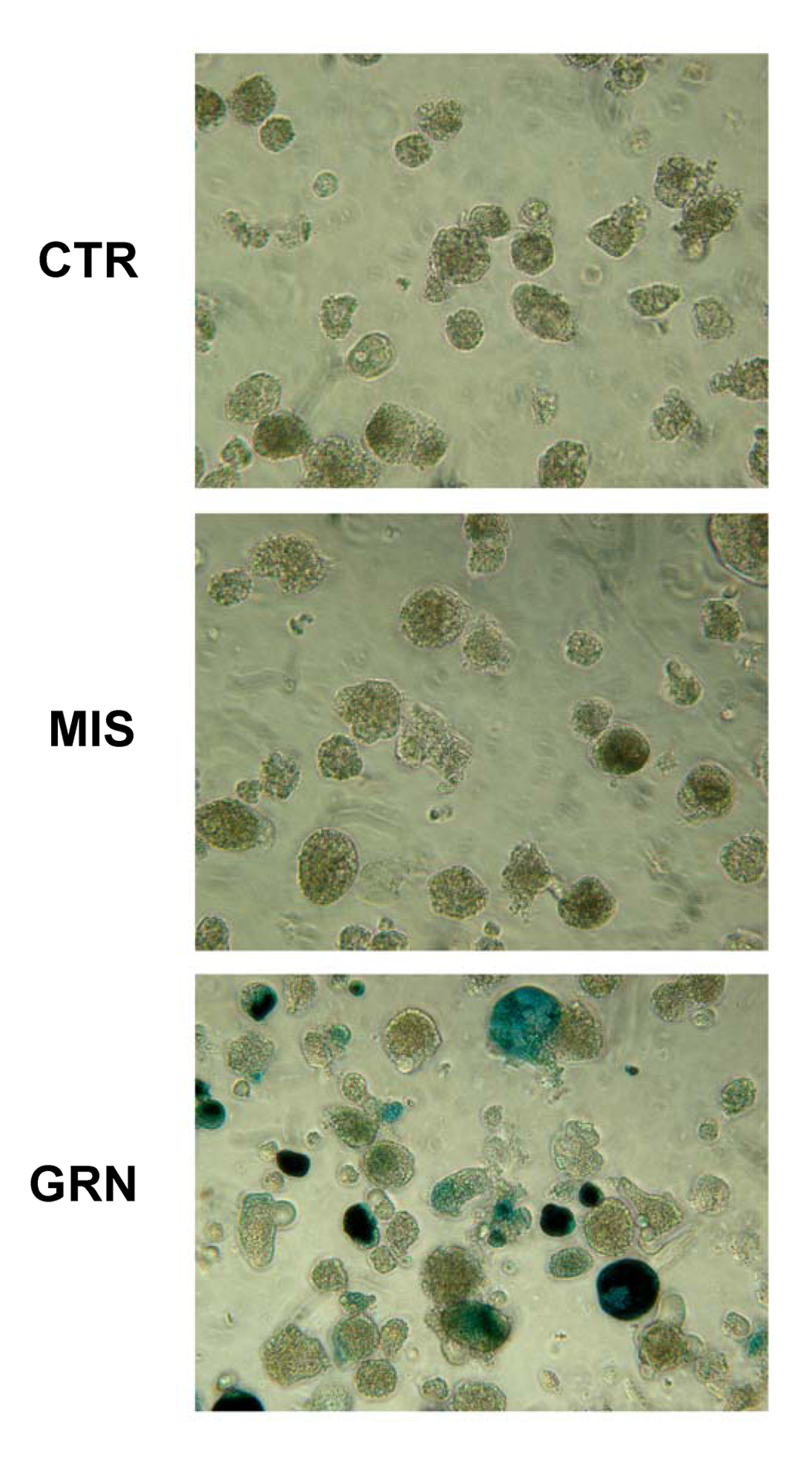

Supplement: Figure S5 — SA-β-galactosidase activity in the floating dead cells harvested from the GRN163L-treated CD18 cells. At the end of the CD18 growth curve, floating cells were collected from the GRN163L-treated (GRN) and control (CTR, MIS) populations. Cells were resuspended in 10 pellet volumes of medium. Suspended cells were fixed, washed, and stained for SA-β-galactosidase activity. Washes before steps were performed by centrifugation. (TIF) [file pone.0085155.s005.tif]
